# The effect of clip material on the retention of implant retained mandibular overdenture: randomized clinical study

**DOI:** 10.1038/s41405-026-00419-y

**Published:** 2026-04-08

**Authors:** Sara Medhat Mohamed, Ahmed M. Sayed

**Affiliations:** 1 Lecturer of removable prosthodontics, Faculty of Oral and Dental Medicine, Modern University for Technology and Information, Cairo, Egypt; 2 Associate Professor of Dental Biomaterials, Faculty of Oral and Dental Medicine, Modern University for Technology and Information, Cairo, Egypt

**Keywords:** Removable prosthodontics, Dental biomaterials

## Abstract

**Aim:**

To evaluate the retention of implant retained mandibular overdenture with polyether-ether-ketone (PEEK) bar using either ready-made nylon clips or custom-made PEEK clips over a one-year randomized clinical study.

**Materials and methods:**

Twenty completely edentulous patients received two implants inserted bilaterally in the canine-premolar region. A milled PEEK bar was screwed to the multiunit abutment screwed to the implants. Patients were divided equally into two groups (*n* = 10) according to clip material; group1: ready-made nylon clip and group 2; PEEK clip (Pressed BioHPP®). Clips were picked up into the fitting surface of the overdenture. Retention force was measured at insertion, three months, six months and one year using universal testing machine. Data were analysed using two-way repeated measure ANOVA followed by Bonferroni post-hoc tests (*p *≤ 0.5).

**Results:**

Retention values between nylon clip group and PEEK group revealed non-statistically significant difference at insertion (28.98 N ± 3.40 and 28.38 N ± 4.80 respectively), three months (30.93 N ± 3.52 and 30.43 N ± 4.7 respectively), six months (30.63 N ± 3.50 and 30.48 N ± 4.63 respectively) and one year (27.13 N ± 3.38 and 27.69 N ± 4.41 respectively).

**Conclusion:**

PEEK clips used for retention of PEEK bar-implant retained mandibular overdentures showed comparable retention values to nylon clips up to one year.

## Introduction

Completely edentulous patients, especially with severely resorbed mandibular ridges, are suffering from denture retention despite the satisfactory constructed dentures. Implant retained mandibular overdenture helps to partly overcome the conventional complete denture limitations such as low retention and stability, progressive bone loss and poor masticatory efficiency. For edentulous mandible, the two-implant retained overdenture is considered the standard option of treatment. It gains its popularity due to its affordability, little invasiveness, relative ease, and increasing quality patient’s life and his satisfaction [1, 2].

For attachment of the overdenture to the underlying implants, many systems have been introduced such as balls/O-ring, bar, clips, sockets and telescopic coping. The bar attachment involves splinting of the implants which resulting in less bone resorption and lower implant loss in comparison to ball retained overdentures. Moreover, the bar retained overdentures provide better masticatory efficiency in comparison to ball retained overdentures [3].

Regarding the bar retained overdenture, it necessitates, at least, 15 mm of an inter-arch distance. Moreover, the optimal length for the bar to accommodate two clips should be between 20 and 22 mm [4, 5] The bar can be constructed from metal such as cobalt-chromium or titanium alloys or from non-metallic materials such as PEEK (Polyether-ether-ketone) and zirconia. However, the retentive clip may be made from metal, PEEK, nylon, Poly-Oxy-Methylene (POM) or zirconia [6, 7].

Unfortunately, the wear of the polymer attachments of the implant supported overdenture became the most occurring complication. The wear of the attachments occurs due to many procedures such as continuous insertion and removal of the overdenture, excessive occlusal load and wrong placement of the overdenture by the patients that resulted from lack of knowledge about the path of insertion. Ciftci et al. [8] (2023) found that 72.6% of the problems that occurred in implant retained overdentures was the wear of the polymeric attachments.

In 1978, PEEK was initially introduced as a thermoplastic, polycyclic, semi-crystalline polymer. It is formed by the grouping of ketone and ether functional groups with aryl rings. PEEK displayed good mechanical properties. Moreover, it reported a good resistance to chemical degradation and hydrolysis. Furthermore, it showed low plaque affinity and no signs of immunogenicity or cytotoxicity that recommend it as a biologically inert material [9].

Consequently, research on attachment retention is crucial for selecting appropriate retention systems tailored to individual patients. The PEEK clip serves as a viable clip material for retention of mandibular retained overdentures. Therefore, many authors compared the performance of PEEK as a clip material with other materials such as nylon [5] cobalt-chromium, zirconia [7] and POM [10] materials and the performance of the PEEK as a clip material reported promising results in comparison with other materials.

Therefore, this study aimed to evaluate retentive force of mandibular overdentures attached to a PEEK bar connecting two implants with two different clip materials; PEEK and nylon for six months. The null hypothesis was that the clip material had no effect in the retention of the implant retained mandibular overdentures.

## Material and methods

### Sample size calculation

The sample size calculation was performed according to repeated measure ANOVA research measured the retention forces of implant supported mandibular overdentures retained by PEEK clip over 12 months follow up period in a randomized clinical trial [11] Partial eta squared was calculated as 0.84, corresponding to an effect size *f* of 2.29. It was calculated with a power of 95% and alpha error probability = 0.05. A total sample size of 4 patients was determined. However, to increase the robustness of the findings and compensate for potential dropouts, the sample size was increased to 20 participants (*n* = 10). Sample size was calculated using G-power V. 3.1.9.7. (Heinrich-Heine-Universität Düsseldorf, Düsseldorf, Germany).

The study design was a randomized parallel group-controlled trial with an allocation ratio of 1:1 and a block randomization. Allocation sequence generation was performed by the second author (using Microsoft Office Excel 2019 (Microsoft Corporation, Redmond, WA, USA)). The second author prepared sequentially numbered, opaque and sealed envelopes containing the group assignments to ensure allocation concealment. Enrolment of participants was performed by the first author where the patients were assessed for eligibility. At the time of bar construction, the first author made each participant to grasp an envelope from a closed box. The first author performed all the treatment steps to minimize operator-related variability. In addition, the first author gave each patient the cleaning and maintenance instructions verbally and written. Briefly, cleansing instructions comprised brushing after meals and before sleeping. In addition, patients were instructed to wear their dentures from 14 to 16 hours per day and remove them during sleeping. Compliance with these instructions was monitored monthly to evaluate the gingival condition by the first author. An independent blinded technician assessed the retention of the dentures.

### Patient selection

Forty-seven patients were screened from the outpatient clinics of the Faculty of Dentistry of MTI University, Cairo, Egypt. Twenty patients were selected according to the following inclusion criteria: completely edentulous male patients with Class I according to the American College of Prosthodontists classification, with a minimum healing period of 6 months following the last tooth extraction, aged 50–60 years, anterior mandibular bone dimensions were at least 13 mm in height and 5 mm in width as assessed radiographically by CBCT, the inter-arch space ranged from 15 mm to 17 mm and free from systemic diseases affecting bone metabolism.

Patients were excluded if they had severe maxillomandibular skeletal discrepancy, presence of para-functional habits, temporomandibular joint disorders, severe bony undercuts, bone metabolic disorders, history of head and neck radiotherapy and intravenous bisphosphonate therapy. In addition, smoking and drug abusing patients were excluded.

The study adheres to CONSORT guidelines. Each participant provided written consent before using their treatment-related radiography data in this study. The implemented radiation protection methods were in accordance with established guidelines.

Every patient made a diagnostic cone-beam computed tomography (CBCT). The CBCT data were obtained and stored as DICOM files (Planmeca Viso G7; Imaging Sciences, Finland) and imported into specialized implant planning software. Virtual planning was carried out by superimposing the CBCT data with a scanned diagnostic cast to determine the optimal implant position. A CAD/CAM surgical guide was then fabricated using 3D printing technology.

### Complete denture construction

Primary impressions of the maxilla and mandible were made using alginate impression material (Zetalgin, Zhermack S.p.A., Italy) then poured to create diagnostic casts. A secondary impression was made using zinc oxide and eugenol impression material (Cavex outline; Cavex R W Holarm, Holland) with special trays. Maxillary and mandibular occlusion blocks were constructed over the master casts consisting auto-polymerizing acrylic resins bases (Acrostone, Cairo, Egypt) and wax rims. The correct vertical dimension of the centric relation in the patient’s mouth was recorded. With a maxillary facebow (Bio-Art Elite Facebow) with Jig Transfer (Panadent Ltd, Cambridge, Ontario, Canada) record for the maxillary cast and a centric jaw relation record made using the wax wafer technique for the mandibular cast, the maxillary and mandibular casts were mounted on a semi-adjustable articulator (Bio-Art articulator, Brazil).

Over the trial denture base, artificial teeth were set. Try-in for the waxed-up denture was carried out. The maxillary and mandibular acrylic dentures were checked in the patient’s mouth for retention, stability, extension and harmonious occlusion after the processing of the waxed-up denture. Any necessary adjustments were done and then the patient was given the denture after receiving the home-care instructions.

### Surgical procedure

For each patient, two implants (Dentis implants, Dentis Co., Ltd., Daegu, Korea) with dimensions (3.2 × 11.5 mm) were inserted bilaterally in the canine-premolar region at an equal distance from the midline, parallel to each other and perpendicular to the occlusal plane. Two-stage surgical protocol was followed for all implants. Following a three-month healing period, covering screws were threaded into the implants.

### CAD/CAM-manufacturing of PEEK bar

During the Prosthetic stage, the implants were left for three months for osseointegration. Following this period, the healing collars were attached to implants after their exposure and left for two weeks for soft tissue healing. In order to screw the multiunit abutments, they were tightened at 30 N torque then retightened two or three times with ten min interval to minimize the settling effect.

An open tray impression technique was performed using a custom-made acrylic resin special tray with window at the implant sites. In order to ensure impression accuracy, a self-cure acrylic resin was used to splint the long transfers copings. This was followed by making a two-stage impression (putty and light body addition silicon impression, KromopanSil, Lascod, Italy). Following impression making, the healing caps were replaced intraorally.

**Fig. 1:**
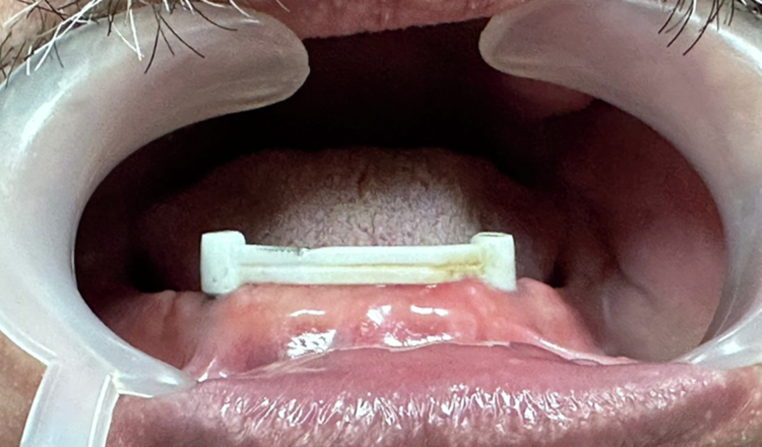
Intraoral view of the screw-retained PEEK bar on the implants.

In order to fabricate the master cast, analogs were screwed to the picked-up impression coping. A verification jig (Duralay, Prestige Dental Products, UK Ltd) was fabricated over the impression coping using self-cured acrylic resin to ensure the accuracy of the master cast. Subsequently, the impression was poured with extra hard dental stone. The passivity was checked intraorally using one screw test. An external desktop scanner was used to scan the master stone cast to produce an STL (Standard Tessellation Language) file that was used for virtual modelling using the CAD/CAM software (Medit 500i.). From the library of the CAD software, the design of the multiunit abutment and bar (Rhein Bar) was selected. The dimensions of the bar were selected according to the study bar design. After the data acquisition, the bar was milled from biological high-performance polymer (BioHPP® BreCAM. Bio-HPP, bredent Gmbh & Co. KG, Senden, Germany) block PEEK type. Multiunit abutments were screwed to the implants and the PEEK bar was screwed to the abutments Fig. 1.

### Patient’s grouping

The patients were assorted into two groups according the clip material. For group I patients (control group), ready-made retentive nylon clips were used for the pickup (Yellow, Medium retention, RHEIN 83. Italy). For group II (test group) patients, retentive clips were fabricated from PEKK material.

### PEEK clip manufacturing

**Fig. 2:**
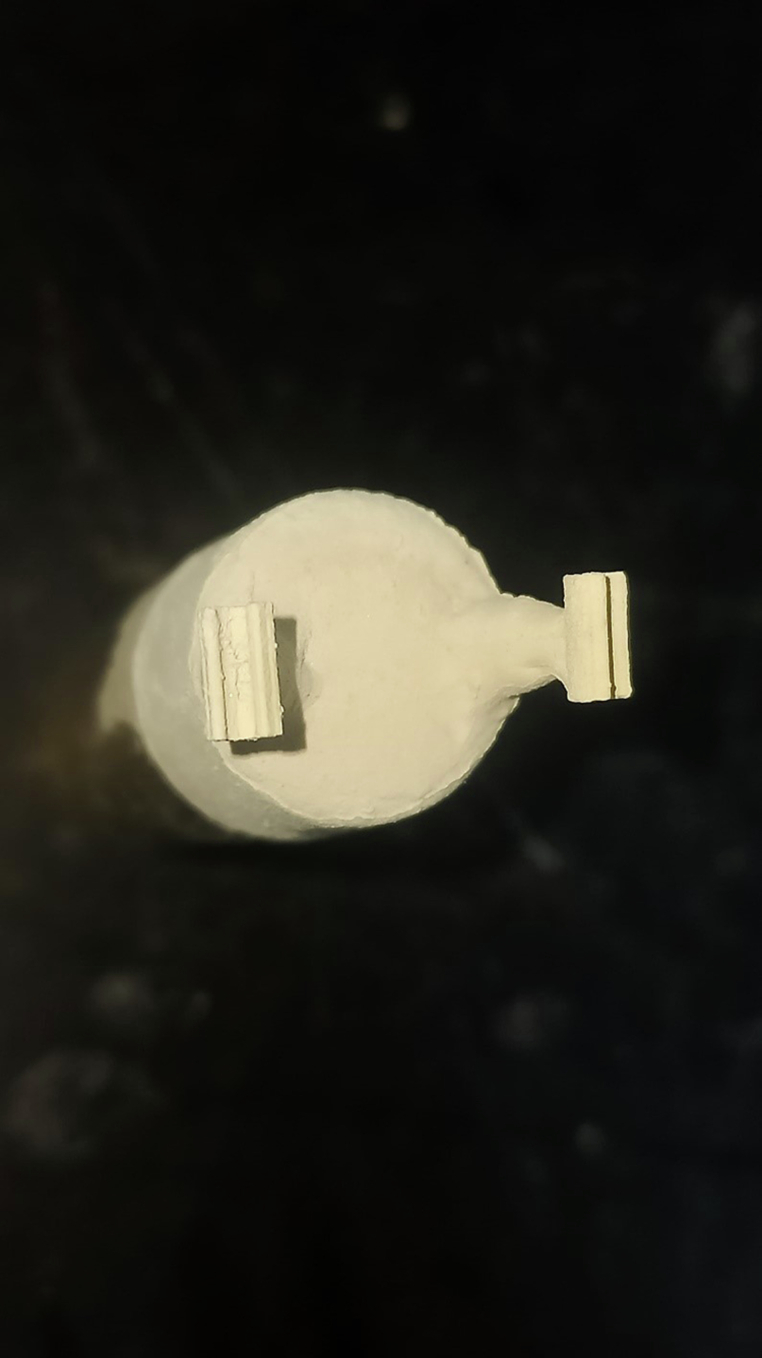
PEEK clips after divesting.

The PEEK clips were manufactured by heat pressing technique of PEEK granules (BioHPP Granulate, Bredent Gmbh & Co. KG, Senden, Germany). The nylon clip was sprued, invested (Ecovest PCS, dent-e-con, Lonsee, Germany) then burnt out (Ibex Apex Three-Stage Burn-Out Oven, USA). According to the manufacturer instructions, the PEEK granules were pressed into the ring using PEEK press machine (for2press, bredent GmbH & Co.KG, Senden, Germany). The PEEK clip was divested using 110 μm sized aluminium oxide particles then cleaned using ultrasonic cleaner for 5 min (Codyson CD-4830, China). (Fig. 2).

### Pick up of denture

**Fig. 3:**
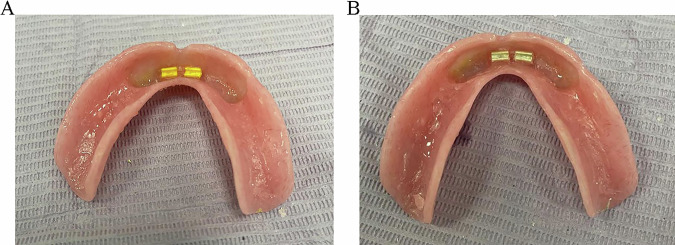
Pick up of the retentive clasps in the intaglio surface of the overdenture. **A** PEEK clip. **B** nylon clip.

The mandibular denture’s fitting surface was examined and marked in the regions opposite and between the implants. After that, the denture’s fitting surface was sufficiently relieved to accept the bars. The retentive clips were picked up by inserting self-cured acrylic resin (Acrostone, Cairo, Egypt) into the relieved areas. After that, the denture was placed in the patient’s mouth until fully seated. The patient was instructed to close in the centric occluding relation with biting force until completely harden the acrylic resin. The denture was then taken out of the patient’s mouth, and any extra material around the clips was removed, finished, polished, and delivered to the patient (Fig. 3).

All the patients were instructed and motivated to maintain their oral and denture hygiene. After denture insertion, the patients were recalled after one week for inspection and making any necessary adjustments.

### Method of measuring retention

**Fig. 4:**
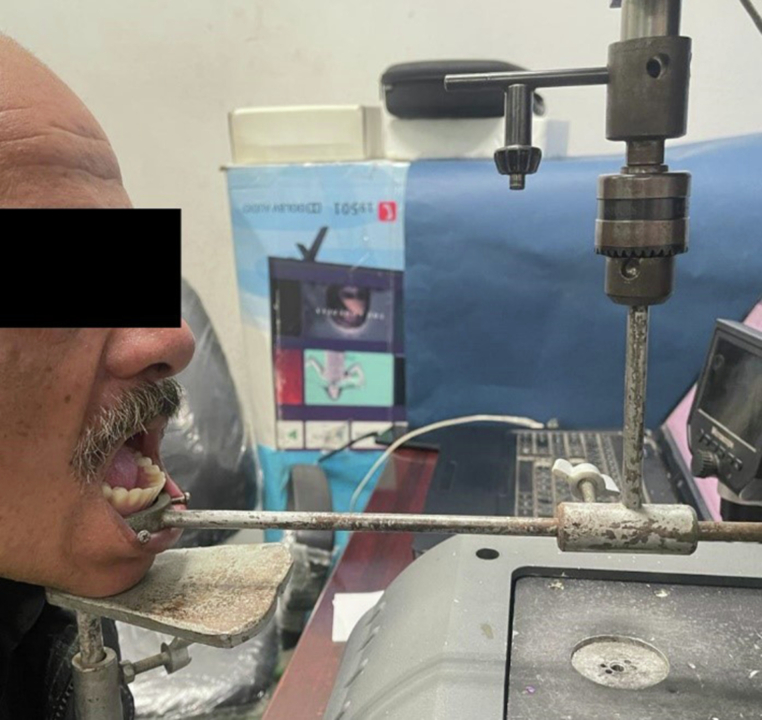
Measurement of the retention force of the mandibular overdenture.

Denture resistance corresponding to vertical displacement force was obtained using a universal testing machine (INSTRON, United States) to measure retention. Each patient had his chin securely fixed on a support while seated upright. The attachment portion of the universal machine was adjusted in accordance with the device’s bar being securely attached to the denture. The device gradually increased the vertical load by 10 mm per minute until the denture completely fell out of place. A tuck sound can be heard to indicate the load at the dislodgment point. Additionally, the force plot in the recorded computer software data exhibits an abrupt drop (Nexyge; Lloyd Instruments; MT-4.6). Each test was repeated five times to obtain five records, and the mean of these records was calculated. Testing was carried out on the mandibular overdenture after insertion, as well as at three months, six months and one year (Fig. 4).

### Statistical analysis

Data was subjected to statistical analysis using IBM® SPSS® statistical version 25 for windows (Armonk, New York, USA). The data were represented as means and standard deviation. Normality and homogeneity of the data were evaluated using Kolmogorov-Smirnov and Levene’s tests. The results showed normal and homogenous distribution. Therefore, two-way repeated measure ANOVA followed by Bonferroni post-hoc tests were used to determine the statistically significant difference between the variables. (*p* < 0.05).

### Ethics declaration

Ethics committee approval for the study was obtained from the Research Ethics Committee (REC) of the Faculty of Dental Medicine, Al-Azhar University, with the reference number 970/6525. Each patient provided written consent before the treatment procedures. Confidentiality and participant privacy were maintained. The research complied with the Declaration of Helsinki of 1975, as revised in 2013.

## Results

Two-way repeated measure ANOVA revelated that the time had a statistically significant effect on the retention values (*p*-value = 0.000). On the other hand, the material reported no statistically significant effect on the retention values (*p*-value=0.943), suggesting comparable overall retention forces between Nylon and PEEK clips. However, the interaction between time and material was recorded a statistically significant effect (*p*-value = 0.023) which indicates that the pattern of retention changes over time differed slightly between Nylon clip and PEEK clip Tables 1, 2.

**Table 1:** The mean, standard deviation (SD) and Confidence Interval (CI) of the retention forces (N) of the two clip materials at different time intervals.

	Pairwise comparison		Nylon	PEEK
After insertion	b	Mean	28.98	28.38
		SD	3.4	4.8
		95% CI	25.201 – 32.766	24.599–32.164
3 months	a	Mean	30.93	30.43
		SD	3.52	4.7
		95% CI	27.155–34.712	26.655–34.212
6 months	a	Mean	30.63	30.48
		SD	3.5	4.63
		95% CI	26.898 – 34.368	26.748–34.218
12 months	c	Mean	27.13	27.69
		SD	3.38	4.41
		95% CI	23.554–30.703	24.114–31.263

Different letters indicate statistically significant difference between the time intervals at *p* ≤ 0.05 using Bonferroni Post-hoc test.

**Table 2:** Tow-way repeated measures ANOVA results.

Effect	Degree of freedom	F value	*P* value	Partial Eta Squared
Time	3	131.917	0.000*	0.930
Material	1	0.005	0.943 NS	0.001
Time x Material	3	3.666	0.023*	0.268

^*^indicates statistically significant difference between data at *p* ≤ 0.05 using two-way repeated measure ANOVA test.

*NS* indicates non- statistically significant difference between data at *p* > 0.05 using two-way repeated measure ANOVA test.

## Discussion

Bar-retained implant-supported overdentures gives acceptable retention and stability specially in patients suffering from flat posterior mandibular residual ridges. Due to the geometrical shape of the bar, it provides transvers support to the covering prosthesis. However, the frequent maintenance of the retentive part of bar attachment system, such as clips, considered a noticeable limitation of the success of that kind of prostheses [2].

The milling of PEEK bars using CAD/CAM technology reduced the laboratory steps, thereby decreasing the risk of distortion and providing the passive fit of the constructed with subsequence of long-term clinical success rates [12]. Nonetheless, the additional cost of CAD/CAM usage in comparison to conventional casting technique is a noticeable disadvantage [6].

The present study demonstrated no statistically significant difference in the retentive forces of overdentures between the two groups at all evaluation periods. Elsayed et al., (2025) in a finite element analysis study, reported a higher deformation in the acrylic overdentures attached to polymeric prosthetic components in comparison to titanium components. This was attributed to the lower modulus of elasticity of the polymeric attachments that produced more strain under occlusal stresses. Therefore, the used PEEK bar in the present study may have resulted in micro-deformation in the mandibular overdenture that could influence stress transmission to the clips. This stress may contribute to comparable wear behaviour of both groups and thereby explaining the absence of significant difference between the two groups [13].

The current study conflicted with other studies regarding the retention forces immediately after insertion [5, 6, 10, 14, 15]. This contradiction may be referred to the difference in the methodology of the current study.

Regarding the in vivo studies; in the present work, the final overdentures were constructed before the implant placement and the first measure of retention was performed after nearly three months of clinical use of the overdenture. However, Emera and Altonbary [10] (2019) and Gamal [14] (2022) constructed the mandibular overdentures and examined the retention forces first time immediately after overdenture construction.

Nevertheless, the increase in retention of the acrylic resin denture with time can be referred to the accentuation of the neuromuscular coordination and other physical factors that affect the denture retention [16–18].

Regarding the invitro studies, the current results were contradicted with other invitro studies that reported higher retention forces with PEEK clips in comparison to nylon or plastic clips [5, 6, 15].

Nassar and Abdelaziz [5] (2022) compared the retention forces of clips made from PEEK and nylon clips. They designed a mandibular cast with two implants connected with PEEK bar, then constructed an overdenture which was retained by two clips.

This could be explained by the presence of other clinical factors rather than the effect of the clip material that affects the denture retention such as presence of saliva and its surface tension, atmospheric pressure, engaging the denture with undercuts and the muscular coordinates of the orofacial muscles [16, 18].

The results of this work recorded a statistically significant decrease in the forces of retention of the overdentures connected to PEEK clips after 12 months. This result is in accordance with the researches done by Emera and Altonbary [10] (2019) and Gamal [14] (2022) as they reported a decrease in the retention of overdentures with PEEK clips after twelve months of clinical use and they referred this decline in retention to the wear of the unfilled PEEK clips used in their studies. They used bars with higher hardness values (zirconia bar, nickel chromium and cobalt-chromium bars respectively) and unfilled PEEK clips with lower hardness values in comparison to PEEK composites. This high difference in hardness value affects the wear of the attachment component with subsequently the retention forces.

Nevertheless, the current work used composite PEEK bar (BioHPP®) with composite PEEK clips. The close approximation of the hardness values of the BioHPP® (30 VHN) and the nylon (25 VHN) may result in lower wear of the PEEK clip. In addition, the PEEK composites reported higher wear resistance than the unfilled PEEK [19].

However, the PEEK composite (BioHPP®) is subjected to mechanical degradation with aging due to contact with saliva and pH fluctuation which result in increasing space between polymeric chains.

Regarding the retention forces of overdentures with nylon clips, the current results showed a statistically significant increase in retention at 3 months and 6 months in comparison to first measuring period then decrease in retention at 12 months. The retention of polymeric clips with time is a controversial issue. Some authors reported a decrease in retention with time [5, 6, 10, 15]. On the other hand, other studies reported an increase in the retention forces with time when using polymeric attachments as a result of deformation of the attachment that results in their hardening or the thermal expansion of the attachment during testing procedures or the saliva composition and the oral environment [20–24].

From the results of the present study, the null hypothesis was partially accepted as the clip material reported no statistically significant difference on the retention forces of the mandibular overdentures.

There are several limitations in the current study. Although the sample size calculation was performed using a priori power analysis, the small sample size and 12-month follow-up may influence the generalizability of the results. The single-center study design may also affect external validity. In addition, patient-reported outcome measures were not assessed, and there was no assessment of laboratory fatigue and mechanical aging.

Future multicenter studies with larger sample sizes and longer follow-up periods are recommended. In addition, collection of patient-reported outcome measures and evaluation of mechanical fatigue are recommended.

## Conclusion

Up to one year, PEEK used as a clip material for bar-clip attachment system for overdentures showed the same retention forces as conventional nylon clips. In addition, its higher cost may be considered a limitation factor.

## Data Availability

The datasets used and/or analysed during the current study are available from the corresponding author on reasonable request.
